# Presepsin and sepsis-induced acute kidney injury treated with continuous renal replacement therapy: will another promising biomarker bite the dust?

**DOI:** 10.1186/s13054-015-1146-8

**Published:** 2015-12-15

**Authors:** Patrick M. Honore, Rita Jacobs, Inne Hendrickx, Elisabeth De Waele, Viola Van Gorp, Herbert D. Spapen

**Affiliations:** ICU Department, Universitair Ziekenhuis Brussel, Vrije Universiteit Brussel, Brussels, 1090 Belgium

A recent meta-analysis published in *Critical Care* proposed presepsin as a novel valuable biomarker for discriminating systemic inflammation from true sepsis [1]. Unfortunately, this excellent paper did not provide information about the occurrence and eventual treatment of acute kidney injury (AKI) in the studied population. The kidney is one of the most frequently afflicted organs during sepsis and AKI may complicate up to half of the cases of blood culture-positive septic shock [2].

Little information is available on presepsin in patients with kidney dysfunction except that levels tend to increase with digressive glomerular filtration rate and are markedly high in patients with chronic renal failure or receiving hemodialysis [3]. More importantly, continuous renal replacement therapy (CRRT) is increasingly used to treat AKI in critically ill patients. C-reactive protein and procalcitonin, which are actually the most commonly used biomarkers to support diagnosis and to evaluate treatment in septic patients, are both significantly eliminated during CRRT [4].

Presepsin is fragmented from a larger glycoprotein and has a molecular weight of approximately 13 kDa. This is of particular concern because it theoretically exposes presepsin to significant convective elimination. Presepsin clearance may even be higher than expected because the molecule may “stick” to the highly adsorptive membranes incorporated in modern CRRT devices [5]. Thus, presepsin cannot be proposed as an accurate and clinically relevant sepsis biomarker until its behavior during CRRT is better specified.

## Abbreviations

AKI: Acute kidney injury
CRRT: Continuous renal replacement therapy
